# BKCa channels regulate the immunomodulatory properties of WJ-MSCs by affecting the exosome protein profiles during the inflammatory response

**DOI:** 10.1186/s13287-020-01952-9

**Published:** 2020-10-15

**Authors:** Ahui Song, Jingjing Wang, Yan Tong, Junyan Fang, Yi Zhang, Huiping Zhang, Hongqiang Ruan, Kai Wang, Yingli Liu

**Affiliations:** 1 Department of Nephrology, Shanghai Ninth People’s Hospital, Shanghai Jiao Tong University School of Medicine, 639 Zhizaoju Road, Shanghai, People’s Republic of China; 2 The Clinical and Translational Research Center Shanghai First Maternity and Infant Hospital, Tongji University School of Medicine, Shanghai, People’s Republic of China; 3 Shanghai Applied Protein Technology Co., Ltd.,Research & Development Center, 58 Yuanmei Road, Shanghai, People’s Republic of China

**Keywords:** BKCa channels, WJ-MSCs, Exosomes, Proteomics, Macrophages

## Abstract

**Background:**

Wharton’s jelly-derived mesenchymal stem cells (WJ-MSCs) from the human umbilical cord have been studied extensively due to their immunomodulatory functions. Large-conductance Ca^2+^-activated K^+^ (BKCa channels) channels are involved in many inflammatory responses, but their involvement in the anti-inflammatory activity of WJ-MSCs is unknown. The underlying molecular mechanism, through which BKCa channels mediate the immunomodulation of WJ-MSC, which may include changes in exosomes proteomics, has not yet been clarified.

**Methods:**

Alizarin staining, Oil Red O staining, and flow cytometry were used to identify WJ-MSCs, which were isolated from human umbilical cord Wharton’s jelly. BKCa channels were detected in WJ-MSCs using western blotting, real-time polymerase chain reaction (real-time PCR), and electrophysiology, and cytokine expression was examined using real-time PCR and enzyme-linked immunosorbent assays (ELISAs). Exosomes were characterized using transmission electron microscopy and nanoparticle tracking analysis. Proteomics analysis was performed to explore exosomal proteomic profiles.

**Results:**

The cells derived from human umbilical cord Wharton’s jelly were identified as MSCs. BKCa channels were detected in the isolated WJ-MSCs, and the expression of these channels increased after lipopolysaccharide (LPS) stimulation. BKCa channels blockade in LPS-treated WJ-MSCs induced apoptosis and decreased interleukin-6 (IL-6) expression. Furthermore, THP-1 cells (human monocytic cell line) stimulated with LPS/interferon gamma (IFN-γ) produced more anti-inflammatory cytokines after treatment with exosomes derived from BKCa channel-knockdown WJ-MSCs (si-exo). We also observed altered expression of mitochondrial ATP synthase alpha subunit (ATP5A1), filamin B, and other proteins in si-exo, which might increase the anti-inflammatory activity of macrophages.

**Conclusions:**

Our study described the functional expression of BKCa channels in WJ-MSCs, and BKCa channels regulated the immunomodulatory properties of WJ-MSCs by affecting the exosomal protein profiles during the inflammatory response.

## Background

Inflammation is defence mechanism against external stimuli. Macrophage activation during inflammation participates in the development of a continuous inflammatory cascade, subsequently resulting in immune imbalance, serious infections, and multiple organ dysfunction, such as sepsis [1–3]. Inflammatory cytokines, including interleukin-6 (IL-6) and tumour necrosis factor alpha (TNF-α), are mainly produced by lipopolysaccharide (LPS)-activated macrophages. The overproduction of IL-6 may result in inflammatory and autoimmune disorders, whereas therapeutic agents targeting the IL-6 axis can effectively treat acute and chronic inflammation, suggesting that IL-6 plays an important role in the human cytokine network. Current treatments for inflammation include antibiotics, neutralizing antibodies against inflammatory factors, and symptomatic support therapy, but their ameliorative effects on severe inflammation remain poor.

Large-conductance Ca^2+^-activated K^+^ (BKCa) channels (also called MaxiK channels) are G protein-coupled transmembrane proteins composed of a core α subunit with or without auxiliary β subunits [4–6]. The physiological function of BKCa channels is to maintain the potassium balance and regulate neurotransmitter release and vascular reactivity [7–9]. An increasing body of evidence has revealed the potential role of BKCa channels in the inflammatory response [10]. However, different models have yielded inconsistent results regarding the function of BKCa channels in inflammatory responses. BKCa channel activation in an asthma model reduced airway inflammation [11], whereas BKCa channel inhibition in a mouse model of pancreatitis decreased the macrophage-mediated inflammatory response [12]. Based on this evidence, BKCa channels might be a potential target of inflammation interventions [10, 12–15].

Mesenchymal stem cells (MSCs) are defined as a cell type with self-renewal ability and multipotent abilities. MSCs have been shown to inhibit immune cell activation, reduce inflammatory factor production, affect B lymphocyte maturation and antibody production, promote immune tolerance, and regulate immune balance; thus, manipulation of these cells is an ideal strategy for the treatment of inflammatory diseases [16–18]. MSCs relieve the inflammatory response by secreting various cytokines and extracellular vesicles, including exosomes [19]. MSCs derived from human umbilical cord Wharton’s jelly (WJ-MSCs) exhibit high plasticity and low immunogenicity, and they can be used in in vitro experiments without ethical approval. Thus, this cell type is a promising candidate for the treatment of inflammatory diseases [20, 21].

Exosomes, which range in size from 50 to 200 nm in diameter, contain proteins, phospholipids, nucleic acids, and other substances secreted by cells; they function as messengers of intercellular communication [19, 22]. As they are formed of phospholipids bilayer, exosomes are not easily destroyed by enzymes and are stable in the blood and urine, as well as supernatant of cultured cells. Exosomes secreted by stem cells can exert excellent therapeutic effects while avoiding the high risks of embolism, tumorigenesis, and immunological reactions associated with direct stem cell injection [23, 24]. MSC-derived exosomes exhibit the immunoregulatory properties of MSCs, and many studies have reported the efficacy of MSC exosomes in the treatment of some pathological conditions, such as osteoarthritis, sepsis, and myocardial inflammation [19, 25–27].

Experimental evidence for the immunomodulatory role of the BKCa channel in WJ-MSCs is still lacking. Zhang et al. found that BKCa channels can inhibit the proliferation of bone marrow mesenchymal stem cells (BMSCs) [28]. In the present study, we explored whether BKCa channels expressed in WJ-MSCs affect the proliferation and immunomodulatory properties of WJ-MSCs by altering the exosomal protein profile during the acute inflammation process.

## Materials and methods

### Cell sources

Umbilical cord samples were collected at the Shanghai First Maternity and Infant Hospital using a procedure approved by the Research Ethics Committee of Shanghai First Maternity and Infant Hospital, and informed consent was obtained from the mothers who donated the umbilical cord specimens.

### Cell isolation, culture, and treatment

After receiving caesarean-delivered full-term female neonates, the umbilical cords were washed several times with phosphate-buffered saline (PBS) to remove the blood, and the umbilical arteries and veins were then removed. Wharton’s jelly was exposed and chopped into several tissue pieces of approximately 1 mm^3^. These small pieces were then transferred into T-75 flasks containing 10 ml of complete medium (minimal essential medium alpha (αMEM, Hyclone, USA) supplemented with 10% foetal bovine serum (FBS, Bioind, Israel) and a 1% penicillin and streptomycin solution) and incubated at 37 °C in an atmosphere with 5% CO_2_ and saturated humidity. After culture for 7–10 days, the cord tissues were removed, and the medium was changed every 3 days thereafter. The morphology of the cultured primary cells was observed under a light microscope (*n* = 3). Once the cultures reached approximately 80–90% confluency, the cells were digested with a 0.25% trypsin-EDTA solution and transferred into another new flask. Passage (P) 3 to P7 WJ-MSCs were treated with different concentrations of LPS (0, 10, 25, 50, and 100 ng/ml) for 24 h, and the expression of BKCa channels was then analysed (*n* = 4).

A human monocytic cell line (THP-1) was purchased from American Type Culture Collection (ATCC) and cultured in RPMI 1640 medium (Roswell Park Memorial Institute 1640, Thermo Fisher, USA) supplemented with 10% FBS (Bioind, Israel) and a 1% penicillin and streptomycin solution (Gibco Company, USA). THP-1 cells (5 × 10^5^ cells/ml) were stimulated with phorbol 12-myristate 13-acetate (PMA, 100 nM, Sigma) for 48 h, and the nonadherent cells were removed. The adherent cells were subsequently incubated with LPS (100 ng/ml, Sigma) and IFN-γ (20 ng/ml, Sigma) for 24 h [29, 30], and then free medium, untreated WJ-MSC exosomes (nc-exo, 100 μg/ml), or BKCa channel-knockdown WJ-MSC exosomes (si-exo, 100 μg/ml) [31] were added to analyse the changes in the cytokine levels (*n* = 4).

### Induction of osteogenic and adipogenic differentiation and identification of WJ-MSCs

WJ-MSC differentiation was induced by incubating P3 WJ-MSCs in six-well culture plates with osteogenic differentiation medium (DMEM supplemented with 10% FBS, a 1% penicillin and streptomycin solution, 1 μM dexamethasone, 10 μM insulin, 0.5 mM 3-isobutyl-1-methylxanthine (IBMX) and 200 μM indomethacin) for 2 weeks. The cells were washed twice with PBS, fixed with 95% ethanol for 10 min, stained with alizarin red for 30 min, and washed with PBS. Photographic images were captured using a microscope (Nikon, Japan) (*n* = 3).

WJ-MSC adipogenic differentiation was induced by incubating P3 WJ-MSCs in six-well culture plates with solution A (Cyagen, USA) for 3 days and adipogenic induction solution B (Cyagen, USA) for 1 day, and then cells were cultured for 21 days before Oil Red O staining. The cells were washed with PBS twice, followed by fixation with 4% paraformaldehyde for 20 min. The cells were washed with PBS before an incubation with the Oil Red O solution for 30 min. Photographic images were captured using a microscope (Nikon, Japan) (*n* = 3).

### Flow cytometry

P3 WJ-MSCs (1 × 10^6^ cells) were digested, washed with PBS by centrifugation at 1000 rpm for 5 min and then suspended in 1 ml of PBS. Subsequently, 100 μl of the cell suspension were transferred into 1.5-ml tubes. One tube was used as a negative control, and the other tubes were incubated with cluster of differentiation 73 (CD73)-fluorescein isothiocyanate (FITC), CD90-FITC, CD105-phycoerythrin (PE), CD34-PE, CD29-FITC, or CD45-FITC antibodies for 30 min. The cells were then analysed using flow cytometry. For the apoptosis analysis, WJ-MSCs cultured with different concentrations of paxilline (0, 100 nM, 1 μM, and 10 μM) were suspended in 100 μl of Annexin V binding buffer, incubated with 5 μl of Annexin V-FITC and 5 μl of propidium iodide (PI) for 15 min at room temperature in the dark, and analysed with a flow cytometer (Beckman CytoFLEX FCM) (*n* = 3). The flow cytometry data were analysed using CytExpert and GraphPad Prism 5 software.

### Western blot analysis

Proteins from WJ-MSCs were isolated using RIPA (Beyotime) and All-In-One buffers (Solarbio, P1260-1). The protein concentrations were determined using a bicinchoninic acid (BCA) protein assay kit (Solarbio, PC0020), and the samples were mixed with sodium dodecyl sulphate polyacrylamide gel electrophoresis (SDS-PAGE) loading buffer (5×, Beyotime, P0015). Total protein (20 μg) from each sample was separated by electrophoresis on 8–10% SDS-polyacrylamide gels and transferred to polyvinylidene fluoride (PVDF) membranes (Bio-Rad, Hercules, CA, USA). The membranes were blocked with 5% skim milk for 2 h and incubated with primary antibodies against BKCa channels (1:1000, Alomone, Israel) and glyceraldehyde phosphate dehydrogenase (GAPDH) (1:1000, Proteintech) at 4 °C for 8–16 h. The membranes were then subjected to three 10-min washes with Tris-HCl-buffered saline (TBS) containing 0.1% Tween 20 and then incubated with a secondary antibody for 1 h at room temperature. The membranes were then washed several times and scanned with a hypersensitive chemiluminescence analyser (Amersham Imager 600). The protein destiny of every band was analysed using ImageJ software (*n* = 4 for each group).

### Real-time PCR

After the aforementioned treatments, total RNA was extracted from WJ-MSCs (*n* = 4) or THP-1 cells (*n* = 4) using TRIzol reagent (Sigma) and then reverse transcribed into cDNAs with the Prime Script RT Master Mix (Takara 036A). The obtained cDNAs were used for real-time PCR with SYBR Premix Ex Taq (Takara 420A). PCR consisted of denaturation at 95 °C for 30 s followed by 40 cycles of 95 °C for 5 s and annealing at 60 °C for 34 s. The products of the amplification of the *KCNMA1* gene using real-time PCR were electrophoretically separated in a 1.5% agarose gel (*n* = 3). The following primer sequences were used:

*GAPDH* forward primer, 5′-ACAACTTTGGTATCGTGGAAGG-3′;

*GAPDH* reverse primer, 5′-GCCATCACGCCACAGTTTC-3′;

*KCNAM1* forward primer, 5′-GGCAGCAGTCTTAGAATGAGTAG-3′;

*KCNAM1* reverse primer, 5′-AAAGCCCACCACATGCGTT-3′;

*IL-6* forward primer, 5′- CCTGAACCTTCCAAAGATGGC-3′;

*IL-6* reverse primer, 5′-TTCACCAGGCAAGTCTCCTCA-3′;

*IL-10* forward primer, 5′-GACTTTAAGGGTTACCTGGGTTG-3′;

*IL-10* reverse primer, 5′- TCACATGCGCCTTGATGTCTG-3′;

*TNF-α* forward primer, 5′-GAGGCCAAGCCCTGGTATG-3′; and

*TNF-α* reverse primer, 5′-CGGGCCGATTGATCTCAGC-3′.

### Electrophysiology

The BKCa channel currents were recorded using the whole-cell patch clamp technique at room temperature (22–25 °C). The external solution contained 150 mM NaCl, 5 mM KCl, 1 mM MgCl_2_, 2 mM CaCl_2_, and 10 mM glucose buffered to pH 7.4 with 10 mM HEPES. The osmolarity of all the solutions was maintained at 300–330 mOsm/l. The patch pipette solution contained 120 mM KCl, 30 mM NaCl, 1 mM MgCl_2_, 0.5 mM CaCl_2_, 5 mM EGTA, 4 mM Mg-ATP, and 10 mM HEPES, pH 7.4. The osmolarity of all the solutions was kept at 280–310 mOsm/l. All the drugs used in the electrophysiological experiments were purchased from Sigma-Aldrich. The membrane currents in a representative MSC were activated by 300-ms voltage steps to between − 60 and + 100 from − 80 mV and then to − 30 mV at 0.2 Hz. In the LPS stimulation assay, the membrane currents were activated by a 300-ms voltage clamp to + 80 from − 80 mV and then to − 30 mV. All whole-cell recordings were performed with an Axopatch 200B and analysed using Digidata 1440A and pClamp10 software (Molecular Devices) (*n* = 6).

### Cell proliferation assay

WJ-MSCs were plated in 96-well plates (1 × 10^4^ cells/well) and separately incubated with 0, 100 nM, 1 μM, and 10 μM paxilline for 24 h. Subsequently, 10 μl of the CCK8 reagent (Rainbio, R1000-2) were added to each well, and the plates were incubated at 37 °C for 4 h. The absorbance was then measured at 450 nm using a microplate spectrophotometer (*n* = 4 for each group).

### Measurement of cytokine production

After the aforementioned treatments, the supernatants of WJ-MSCs and THP-1 cells were diluted appropriately, and the levels of IL-6, IL-10, and TNF-α were measured using ELISA kits (all from Biolegend) and a multimode microplate reader (Biotek SynergyH1). The minimum threshold concentrations of the IL-6, TNF-α, and IL-10 proteins detected by each ELISA kit were 7.8, 7.8, and 3.9 pg/ml, respectively (*n* = 4 for each group).

### Cell transfection

Three small interfering RNAs (si1, si2, and si3) targeting the *KCNMA1* gene that encodes the BKCa channel were synthesized by JiMan Biological (China). WJ-MSCs were transfected with the three different BKCa channel siRNAs using Lipo8000 (Beyotime Biotechnology, China), and the transfection efficiency was verified by detecting the levels of the BKCa channel protein and mRNA in the transfected cells (*n* = 4 for each group). The following siRNA sequences were used:

si1 forward (5′ → 3′), GUCUUAGAAUGAGUAGCAA;

si1 reverse (5′ → 3′), UUGCUACUCAUUCUAAGAC;

si2 forward (5′ → 3′), UCACUGAACUAGUGAACGA;

si2 reverse (5′ → 3′), UCGUUCACUAGUUCAGUGA;

si3 forward (5′ → 3′), AAUUGGAAAGAAGGUGAUG; and

si3 reverse (5′ → 3′), CAUCACCUUCUUUCCAAUU.

### Exosome separation, identification, and uptake

After 48 h of culture with exosome-free FBS, WJ-MSC supernatants were obtained from the control group and the BKCa channel-knockdown group and centrifuged at 800 g for 5 min at room temperature. The supernatants were filtered through a 0.22-μm filter and then centrifuged for another 10 min at 2000*g*, and the precipitate was discarded. The supernatants were centrifuged twice for 2 h at 100,000*g* in an ultracentrifuge (Optima xe-90, Beckman Coulter). The obtained sediment (exosomes) was resuspended in PBS for the subsequent experiments. The protein concentrations of the exosomes were quantified using a BCA protein assay kit (Solarbio, China) and identified based on the levels of the marker proteins CD9, CD63, and CD81 using western blotting (all the antibodies were obtained from SBI, System Biosciences Company, USA), transmission electron microscopy (TEM, Tecnai G2 SpiritBiotwin) and nanoparticle tracking analysis (NTA) (Zeta View) (*n* = 4 for each group).

The obtained exosomes were labelled with PKH67 (Sigma) for 4 h and washed with PBS to remove the excess dye. After induction and adhesion, THP-1 cells were incubated with the labelled exosomes for 24 h and stained with DAPI to visualize the nuclei. The localization of exosomes (green) and nuclei (blue) in the stained THP-1 cells was detected using fluorescence microscopy (*n* = 4 for each group).

### SP3 digestion of exosome proteins

Single-pot, solid phase-enhanced sample preparation (SP3) was applied to digest the exosome proteins of 8 samples including 4 nc-exo and 4 si-exo. The exosome proteins (50 μg) for each sample were denatured with 100 μl of lysis buffer (4% SDS and 50 mM Tris-HCl, pH 7.5) and reduced with 10 mM dithiothreitol (DTT) for 40 min at 37 °C. Subsequently, the proteins were alkylated with 25 mM iodoacetamide for 30 min in the dark and quenched with 50 mM DTT for 20 min. A 1:1 mixture of two different types of carboxylate-functionalized beads (Sera-Mag Speed Beads, GE Life Sciences, cat. nos. 45152105050350 and 65152105050350) were added to achieve an estimated concentration ratio of 1:10 (μg of protein/μg of SP3 beads) [32]. Ethanol was added to achieve a specific final concentration (v/v 1:1) for protein binding, and the tubes were incubated at room temperature for 10 min with mixing at 900 rpm in a ThermoMixer. The tubes were placed in a magnetic rack and incubated for 2 min. The supernatant was discarded, and the beads were rinsed three times with 180 μl of 80% ethanol. For elution, the tubes were removed from the magnetic rack, and the beads were resuspended in 100 μl of 25 mM ammonium bicarbonate. Appropriate amounts of trypsin (enzyme to protein ratio of 1:50) (Promega, cat. no. V5071) and Lys-C (1:100 enzyme to protein ratio) (Wako, cat. no. 125-05061) were added to the resuspension buffer, and digestion was performed overnight at 37 °C in a ThermoMixer at 900 rpm. The tubes were placed on a magnetic rack, and the supernatant was recovered and centrifuged at 4 °C for 10 min. The last supernatant was desalted and analysed using liquid chromatography tandem mass spectrometry (LC/MS-MS). The iRT peptides (Biognosys, Schlieren, Switzerland) were spiked into the sample prior to analysis according to the manufacturer’s instructions (*n* = 4 for each group).

### LC/MS-MS and data analysis

The DIA analysis was performed using a mass spectrometer (Q Exactive HFX, Thermo) coupled with a nanoLC1200 system (Thermo) [33, 34]. LC separation was performed using a homemade analytical column with an integrated spray tip (75-μm i.d. × 25 cm) packed with 1.9-μm/120-Å ReproSil-Pur C18 resin (Dr. Maisch GmbH, Germany) at a flow rate of 250 nl/min. The buffers used for separation were 0.1% (v/v) FA in water (buffer A) and 0.1% (v/v) FA in 84% ACN (buffer B). The peptides were separated with a 120-min gradient as follows: 5–8% buffer B for 2 min, 8–23% buffer B for 80 min, 23–45% buffer B for 26 min, 45–100% buffer B for 2 min, and a 10-min wash with 100% buffer B. For MS acquisition, variable isolation window DIA methods with 30 windows were applied. The optimized method involves one full scan and 30 variable window DIA scans. The full scan was set at a resolution of 120,000 over the m/z range of 350 to 1650 followed by DIA scans with the following parameters: resolution, 30,000; NCE, 25%; AGC target, 3e6; and maximal injection time, auto. The cycle time was 3 s.

All the DIA raw files were analysed using Spectronaut 12.0 Pulsar (Biognosys) with the default settings to generate a spectral library for DIA analysis. In brief, the retention time prediction was set to dynamic iRT based on a correction factor of 1. Interference correction at the MS2 level was allowed. Cross-run normalization was enabled to correct for systematic variance in the LC/MS performance, and a local normalization strategy was used. The normalization was based on the assumption that similar average numbers of peptides were up- and downregulated and that the majority of the peptides within the sample were not regulated across runs and along the retention time. All the results were filtered based on a Q value cutoff of 0.01 (corresponding to an FDR of 1%). The peptide intensity was calculated by summing the peak areas of their respective fragment ions for MS2, and the protein intensity was calculated by summing the intensity of the respective peptides.

According to the quantification results, the differentially expressed proteins between the two groups (fold change > 1.5 and *p* value< 0.05) were used for further bioinformatic analyses, which were performed using Blast2Go (https://www.blast2go.com/) software. The categorical annotations were based on KEGG pathways, keywords (UniProt), and Gene Ontology (GO) terms (biological process (BP), molecular function (MF) and cellular component (CC)). All annotations were extracted from the UniProt database (*n* = 4 for each group).

### Statistical analysis

All the results are presented as the mean ± S.E.M. from three independent experiments. The data were analysed using GraphPad Prism 5 software, and differences were considered significant if *p* < 0.05. The statistical significance of the differences in parametric data among more than two groups was determined using one-way ANOVA with Tukey’s post hoc analysis, and the *T* test was used to analyse two unpaired groups.

## Results

### Propagation, identification, and differentiation of WJ-MSCs

**Fig. 1:**
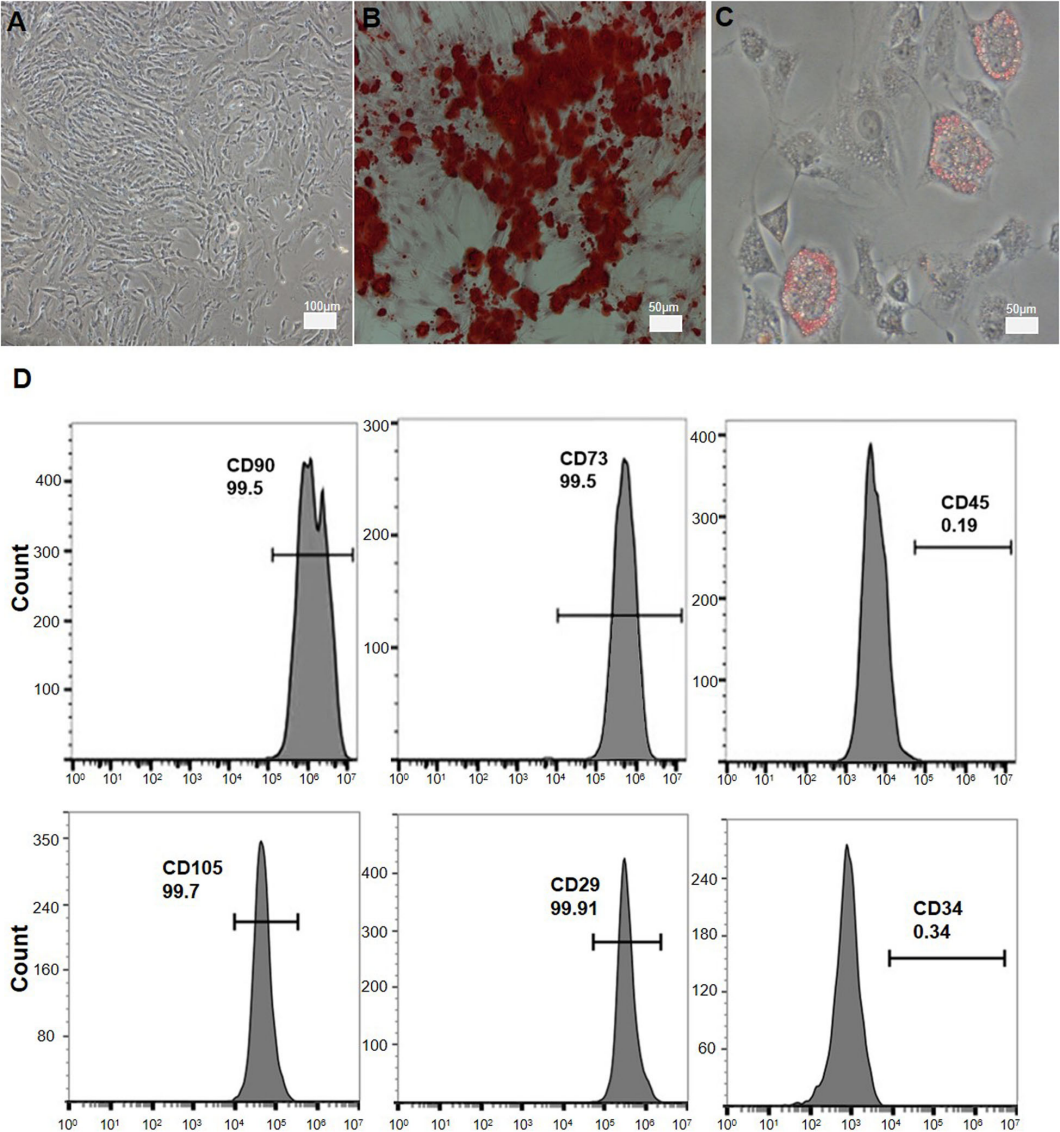
Propagation, identification, and differentiation of WJ-MSCs. **a** Morphology of primary MSCs derived from human umbilical cord Wharton’s jelly using light microscopy. **b** Alizarin red staining for the osteogenic differentiation ability of WJ-MSCs. **c** Oil Red O staining for the adipogenic differentiation of WJ-MSCs. **d** Analysis of the immunophenotypic surface markers (CD90, CD73, CD34, CD29, CD45, and CD105) expressed by WJ-MSCs by flow cytometry

Based on methods described in the literature [35, 36], we used and improved the published tissue block adherence method to successfully obtain WJ-MSCs. After the first passage, WJ-MSCs grew rapidly and exhibited a fibroblast-like morphology, as determined by light microscopy (Fig. 1a). The differentiation capacity of the third passage of WJ-MSCs was detected using alizarin red staining, which revealed some brown calcium deposits (Fig. 1b). WJ-MSCs also displayed Oil Red O-positive lipid droplet deposition (Fig. 1c). We detected surface markers of the third passage of WJ-MSCs using flow cytometry. The WJ-MSCs were strongly positive for CD90, CD29, CD105, and CD73, negative for CD45, and CD34 (Fig. 1d). These characteristics were consistent with the criteria for the identification of MSCs, and differences in these physiological features were not observed among our primary WJ-MSC lines.

### Functional BKCa channel expression in WJ-MSCs

**Fig. 2:**
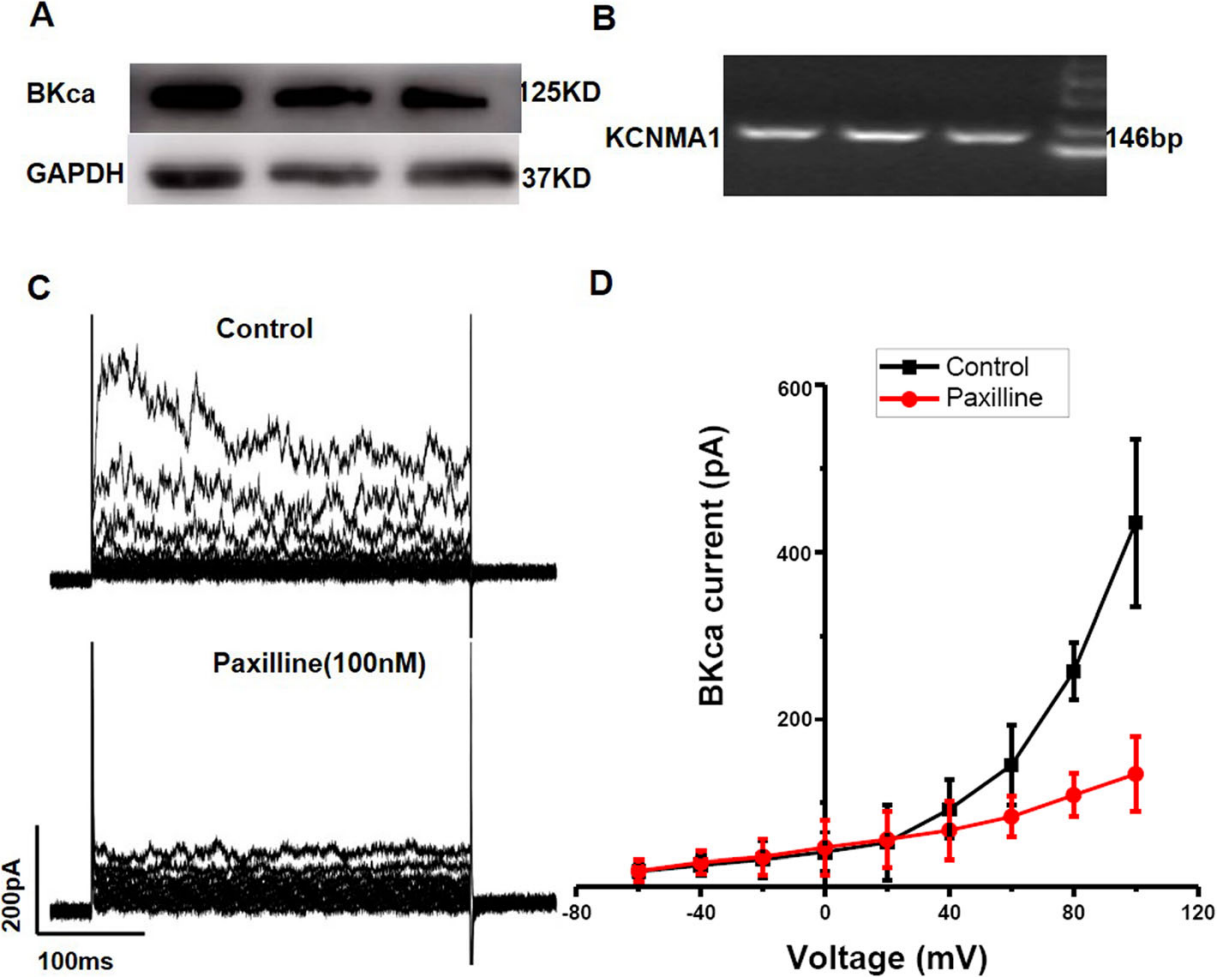
WJ-MSCs express functional BKCa channels. **a** Western blot for BKCa channel protein expression. **b** Agarose gel electrophoresis image of *KCNMA1* amplified from WJ-MSCs with specific primers by real-time PCR. **c** Traces of whole-cell recordings of BKCa channels in WJ-MSCs treated with or without 100 nM paxilline. **d** Membrane currents were activated by 300-ms voltage steps to between − 60 and + 100 from − 80 mV and then to − 30 mV (as shown in the inset). The I-V relationships of the currents under the control conditions and after paxilline treatment are shown

Western blotting was performed to examine the expression of BKCa channels in our primary WJ-MSCs, and the results confirmed their expression in the three different WJ-MSC lines (Fig. 2a). Furthermore, the real-time PCR analysis showed that the WJ-MSCs expressed *KCNMA1* gene, which encodes BKCa channels (Fig. 2b). Whole-cell patch clamp recordings verified that the BKCa channels in WJ-MSCs were functional. We recorded representative traces of the BKCa channels in cultured WJ-MSCs; the currents were inhibited by 100 nM paxilline, a BKCa-specific blocker [37, 38] (Fig. 2c, d). Our results demonstrated the functional expression of BKCa channels in WJ-MSCs.

### LPS induces expression of the BKCa channel mRNA and protein

**Fig. 3:**
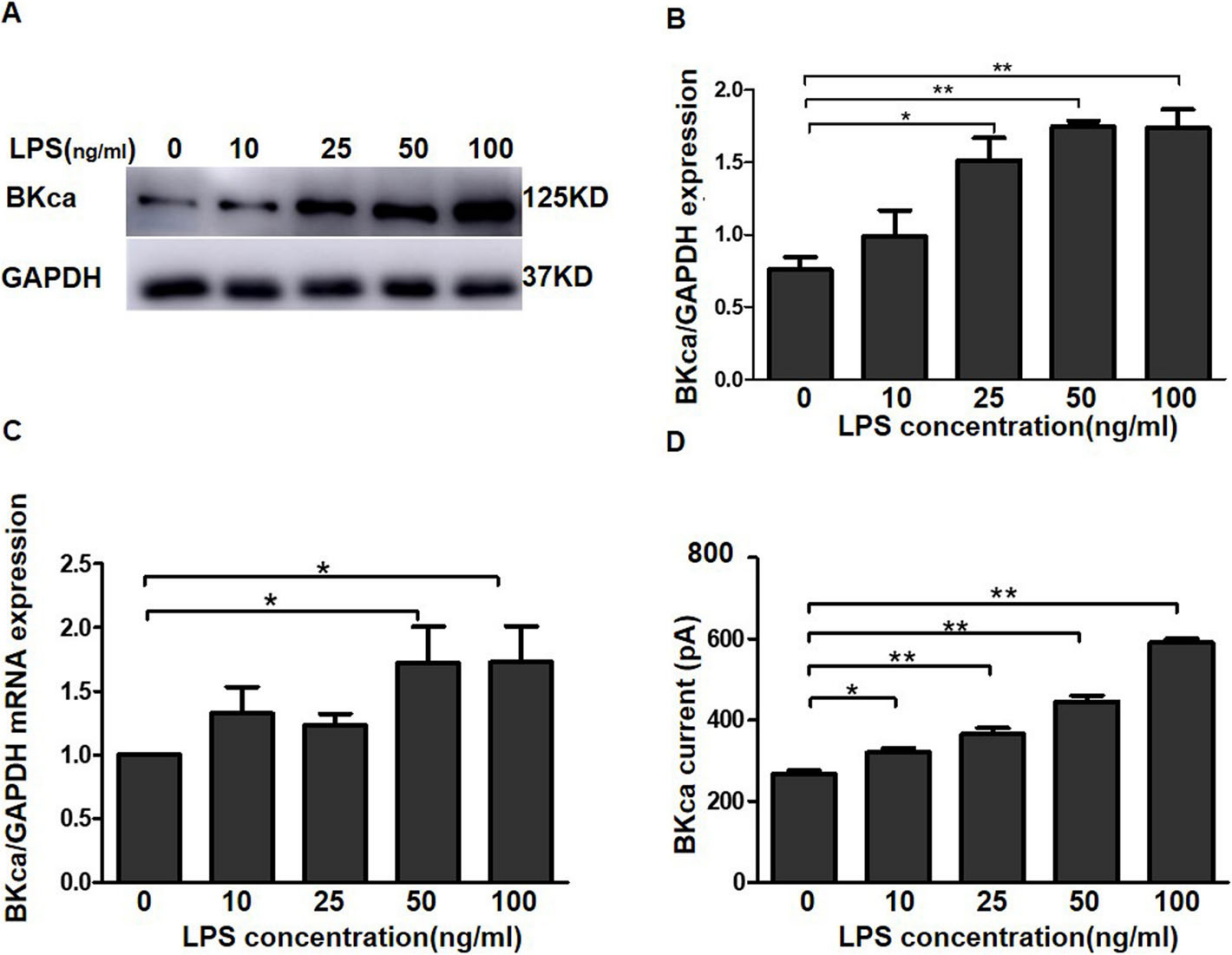
LPS increases the levels of the BKCa channel mRNA and protein expression. **a**, **b** Western blot show BKCa channel protein expression. **c** The expression of the BKCa channel mRNA was analysed using real-time PCR. The results were normalized to the GAPDH level and shown as relative changes. **d** The function of BKCa channels was detected using a voltage patch clamp, which was activated by 300-ms voltage clamp steps to + 80 V from − 80 mV and then to − 30 mV. The data are presented as the mean ± S.E.M. from three independent experiments. The value 0, 10, 25, 50, and 100 represent the corresponding LPS concentrations (ng/ml). *p < 0.05 and **p < 0.01 compared with the 0 group

Immunoblots showed an increase in the levels of the BKCa channel protein in WJ-MSCs treated with LPS (50 or 100 ng/ml) (Fig. 3ab). Real-time PCR was performed to detect the levels of the BKCa channel mRNA after treatment with different LPS concentrations. The levels of the BKCa channel mRNAs increased after LPS treatment (50 and 100 ng/ml), but this effect was not dose-dependent (Fig. 3c). We used electrophysiology to examine whether the BKCa channels expressed at higher levels in WJ-MSCs were functional, and the BKCa channel currents increased after LPS stimulation (Fig. 3d), suggesting that the LPS treatment increased BKCa channel expression and function in WJ-MSCs.

### BKCa channels in WJ-MSCs induce inflammatory cytokine expression

**Fig. 4:**
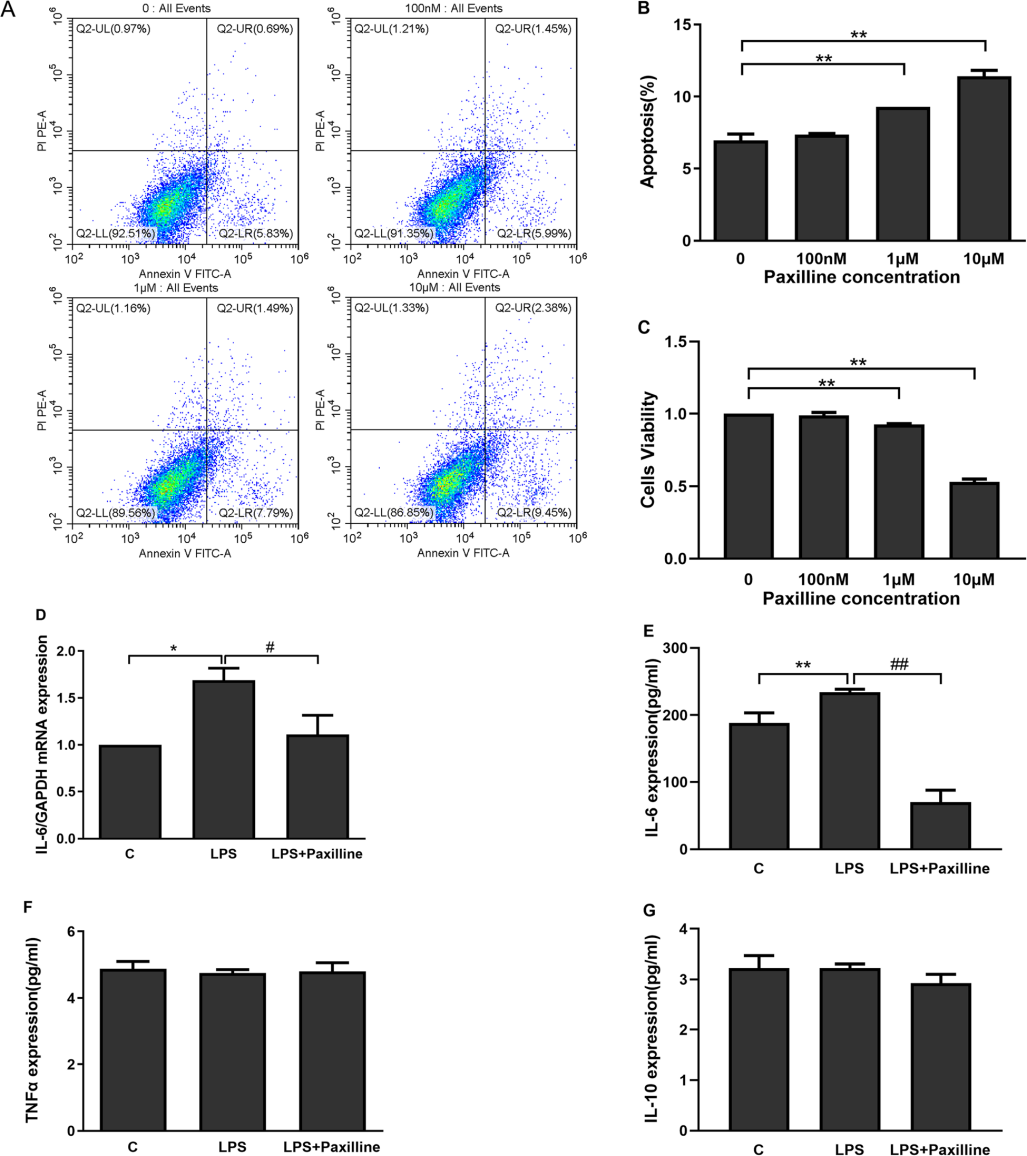
BKCa channels in WJ-MSCs induce inflammatory cytokine expression. **a**, **b** The percentage of apoptotic WJ-MSCs was analysed using flow cytometry. **c** The viability of WJ-MSCs was determined by measuring the absorbance at 450 nm using a microplate reader. WJ-MSCs were stimulated with LPS (50 ng/ml) alone or in combination with paxilline (100 nM) for 24 h. **d**, **e** The expression of the IL-6 mRNA was analysed using real-time PCR (**d**), and the IL-6 protein concentration was measured using an ELISA (**e**). **f**, **g** The TNF-α (**f**) and IL-10 (**g**) concentrations were measured using ELISAs. The data are presented as the mean ± S.E.M. from three independent experiments. *p < 0.05 and **p < 0.01 compared with the C group or 0 group; ^#^*p* < 0.05 and ^##^*p* < 0.01 compared with the LPS group

Paxilline selectively blocks and effectively inhibit BKCa channels in various cells [37, 39]. The flow cytometry results showed higher percentage of apoptotic cells after treatment with high concentrations of paxilline (≥ 1 μM) (Fig. 4a, b). Moreover, high paxilline concentrations (≥ 1 μM) reduced the viability of WJ-MSCs (Fig. 4c).

We detected the expression of inflammatory cytokines to examine the involvement of BKCa channels in WJ-MSCs in inflammation. The real-time PCR and ELISA results showed increased levels of the IL-6 mRNA and protein in LPS-stimulated WJ-MSCs (Fig. 4d, e), which were inhibited by treatment with the BKCa channel blocker paxilline (100 nM). However, the levels of TNF-α and IL-10 in LPS-treated WJ-MSCs were not altered by paxilline, in contrast to its effects on macrophages (Fig. 4f, g).

### Identification of exosomes derived from WJ-MSCs

**Fig. 5:**
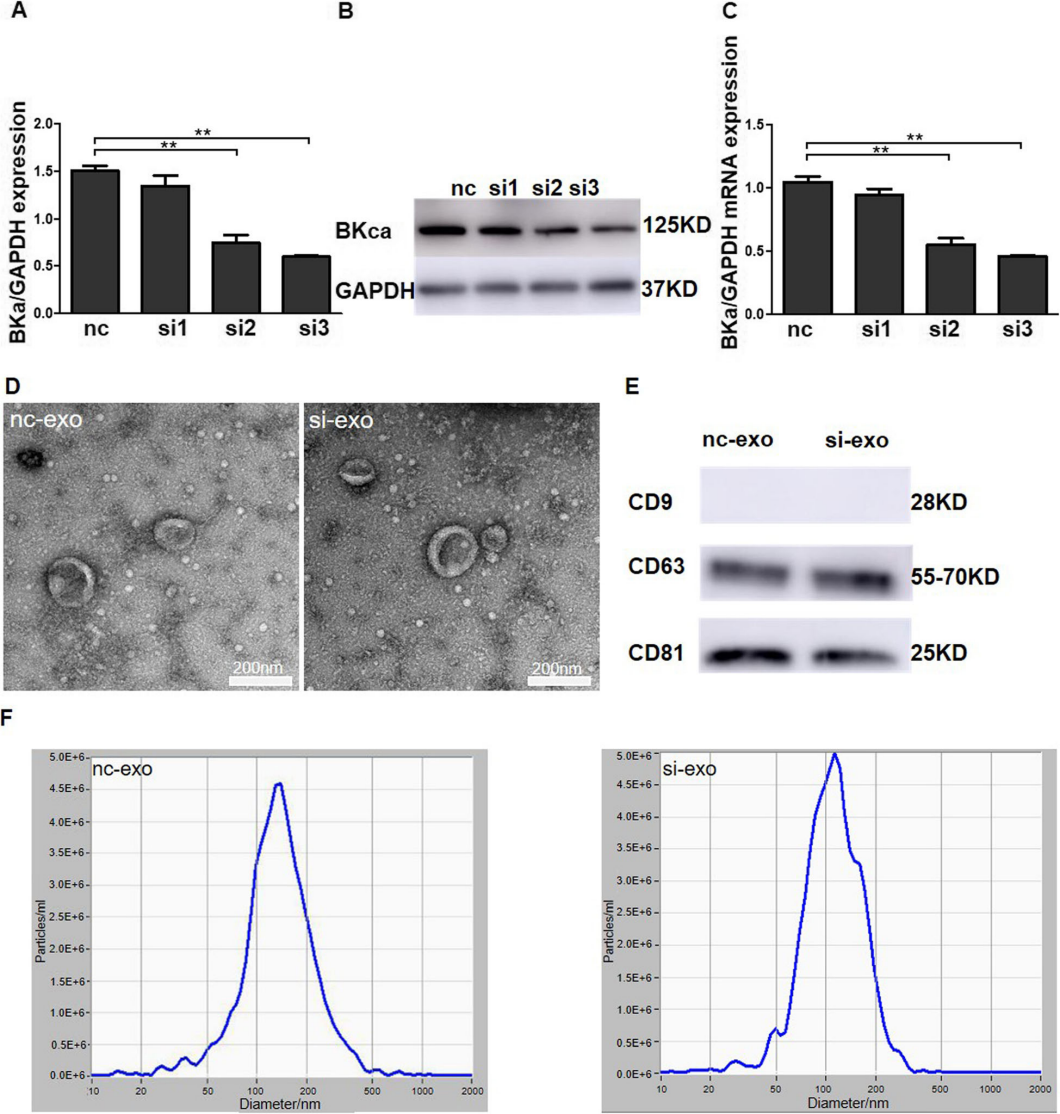
Identification of exosomes derived from WJ-MSCs. **a**, **b** Western blots of the levels of the BKCa channel protein in WJ-MSCs after transfection with the three siRNAs. **c** Real-time PCR detection of the changes in BKCa channel mRNA expression in WJ-MSCs after transfection with the three siRNAs. **d** The morphology of the exosomes from the nc-exo and si-exo groups was viewed using TEM. **e** The levels of CD9, CD63, and CD81 was detected using western blotting. **f** The size distributions of the exosomes from the two groups were analysed using NTA. The data are presented as the mean ± S.E.M. from three independent experiments. *p < 0.05 and **p < 0.01 compared with the nc group

Stem cell therapy inhibits inflammation through exosomes [19, 25–27]. Here, we determined if BKCa channels are involved in the anti-inflammatory activity of WJ-MSC exosomes. First, we used siRNA to knockdown the expression of BKCa channels in WJ-MSCs. The mRNA and protein levels of BKCa channels were significantly decreased by si3 (Fig. 5a–c); thus, we selected this siRNA for use in further experiments. Second, we purified and identified the exosomes derived from WJ-MSCs, and electron microscopy revealed that the exosomes in the nc-exo and si-exo groups exhibited similar cup shapes and diameters (Fig. 5d). Western blot analysis showed that the exosomes from both groups expressed the markers CD63 and CD81, but not CD9 (Fig. 5e). The NTA results revealed similar particle size distributions for the exosomes from the nc-exo and si-exo groups, mainly from 50 to 200 nm (Fig. 5f).

### Exosomes derived from WJ-MSCs modulate inflammation via BKCa channels

**Fig. 6:**
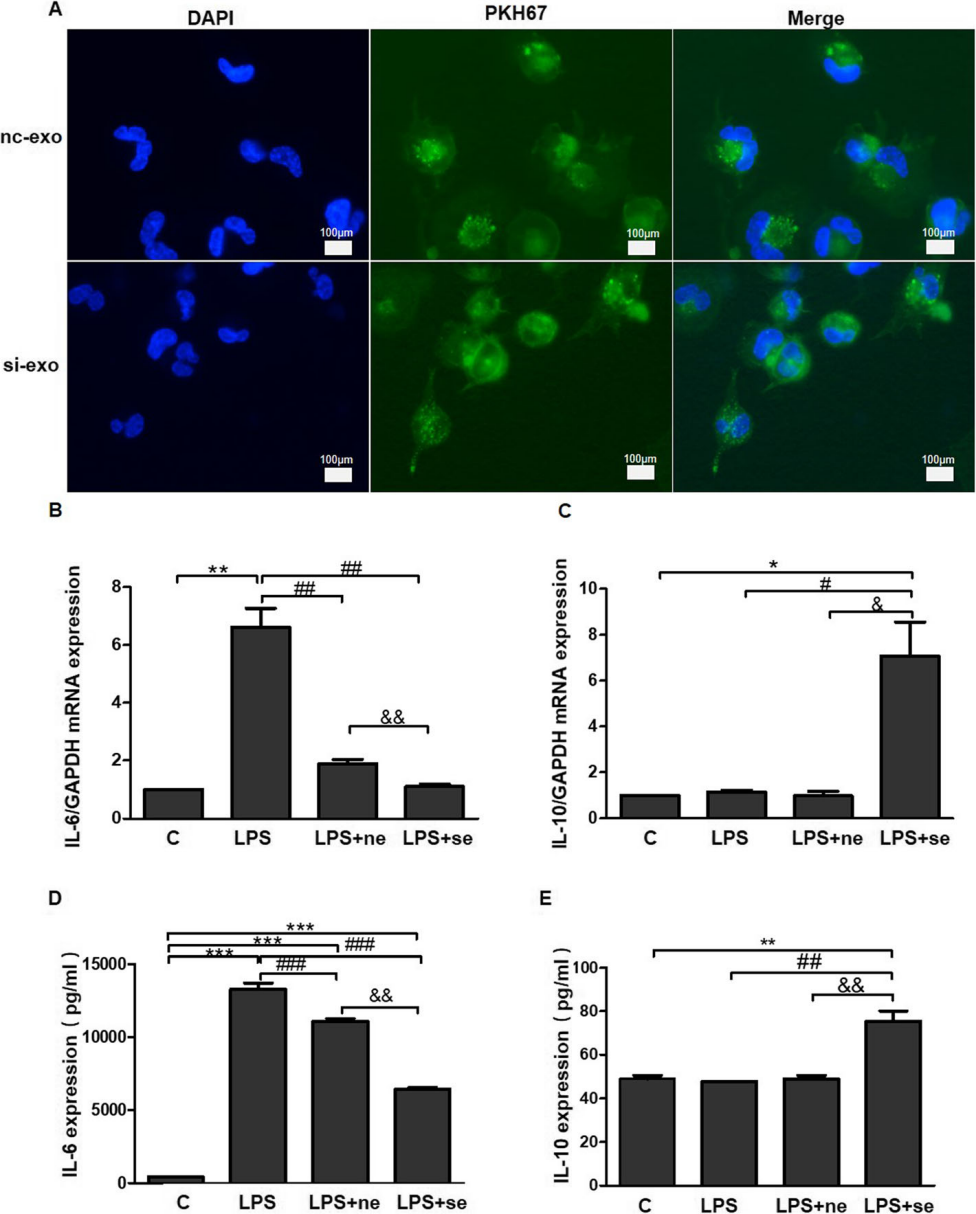
Exosomes derived from WJ-MSCs modulate inflammation via BKCa channels. **a** The colocalization of exosomes with the nuclei (DAPI, blue) of THP-1 cells incubated with PKH67 (green)-labelled exosomes was determined using fluorescence microscopy. **b**, **e** The expression ofIL-6 (**b**) and IL-10 (**c**) mRNAs was detected using real-time PCR, and the levels of the IL-6 (**d**) and IL-10 (**e**) proteins were determined using ELISAs. The data are presented as the mean ± S.E.M. from three independent experiments. *p < 0.05, **p < 0.01, and *** *p* < 0.001 compared with the C group. ^#^*p* < 0.05, ^##^*p* < 0.01, and ^###^*p* < 0.001 compared with the LPS group. ^&^*p* < 0.05, ^&&^*p* < 0.01, and ^&&&^*p* < 0.001 compared with the LPS+ne group

THP-1 cells were used to assess the anti-inflammatory properties of WJ-MSC- derived exosomes. First, exosome phagocytosis in THP-1 cells was examined. Different exosomes were labelled with PKH67 (green) and co-incubated with THP-1 cells, and the results showed that exosomes labelled with PKH67 (green) were positioned around the DAPI-stained nuclei (blue), indicating that both exosome types were absorbed by THP-1 cells (Fig. 6a). Second, the following four groups were included in the experiment to compare the immunomodulatory effects of WJ-MSC-secreted exosomes before and after BKCa channel knockdown: control group (C), LPS stimulation alone group (LPS), LPS stimulation combined with nc-exo group (LPS+ne), and LPS stimulation combined with si-exo group (LPS+se). The expression of the IL-6 mRNA was increased by approximately 6-fold in the LPS group compared with the C group (*p* < 0.01). The IL-6 mRNA levels decreased by 70% and 86% in the LPS+ne and LPS+se groups, respectively, compared with the LPS group (*p* < 0.01). The IL-6 mRNA level in the LPS+se group was decreased by 50% compared with that in the LPS+ne group (*p* < 0.01) (Fig. 6b). The IL-10 mRNA level showed no changes among the control, LPS, and LPS+ne groups, but was increased by 6-fold in the LPS+se group compared with the other three groups (*p* < 0.05) (Fig. 6c). The ELISA results showed a 4-fold increase in the IL-6 concentration in the supernatant of the LPS group compared with the supernatant of the control group (*p* < 0.01). The IL-6 concentration in the supernatant of the LPS+ne and LPS+se groups was decreased by 17% and 50%, respectively, compared with the LPS group (*p* < 0.01). The IL-6 concentration in the supernatant of the LPS+se group was decreased by 42% compared with the supernatant of the LPS+ne group (*p* < 0.01) (Fig. 6d). The IL-10 concentration in the supernatant did not show significant differences among the control, LPS, and LPS+ne groups, but the IL-10 concentration in the supernatant of the LPS+se group was increased by 40% compared with the other three groups (*p* < 0.01) (Fig. 6e).

### Proteomic analysis

**Fig. 7:**
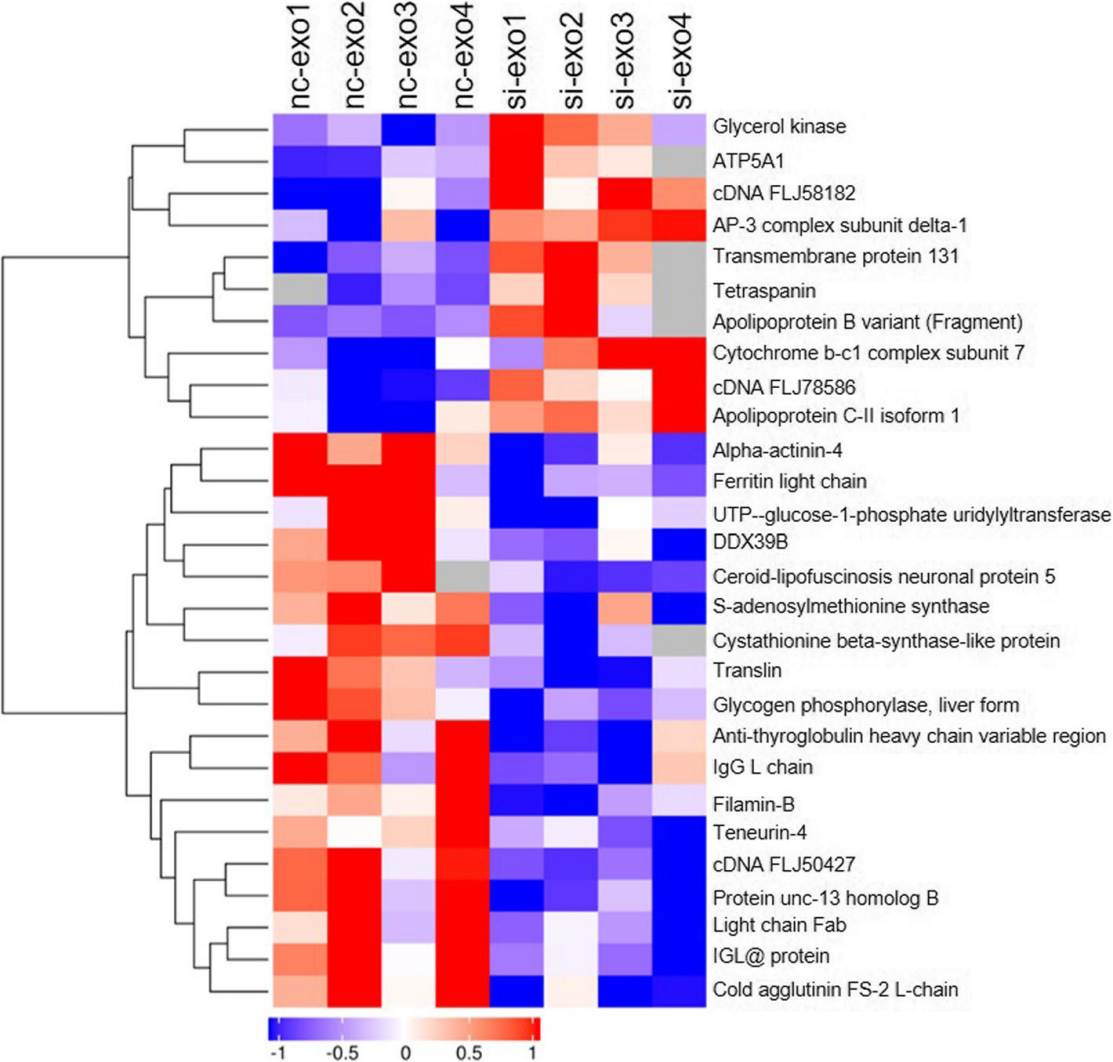
Proteomics analysis. Comparison of the proteins in nc-exo and si-exo groups identified 28 differentially expressed proteins that were significantly enriched in the pathways shown in the heat maps

A total of 2196 protein groups were quantified, and 28 protein groups showed differential expression between the exosomes from the nc-exo and si-exo groups (Fig. 7). The data were subjected to GO and KEGG pathway analyses using Panther and Gene Ontology algorithms and classified based on biological processes and molecular functions (Supplement 1). In the biological process category, the most enriched clusters identified were sequestering of metal ion, generation of precursor metabolites and energy, glucan metabolic process, cellular glucan metabolic process, and glycogen metabolic process (Supplement 1A). In the molecular function category, the proteins related to glucose binding, vitamin B6 binding, pyridoxal phosphate binding, transferase activity, and diacylglycerol binding exhibited the greatest enrichment (Supplement 1A). In the cellular component category, the proteins related to the inner mitochondrial membrane protein complex, mitochondrial protein complex, mitochondrial membrane part, and mitochondrial inner membrane displayed the greatest enrichment (Supplement1A). The KEGG pathways related to lysosomal metabolism, iron metabolism, glycogen and energy metabolism, and oxidative phosphorylation were significantly altered (Supplement 1B). Finally, the expression of proteins related to immunoregulation, including the ATPase α subunit, tetraspanin, and apolipoprotein C-II isoform 1, was significantly higher in the si-exo group. Other proteins, including ferritin light chain, filamin B, and translin, were expressed at lower levels in the si-exo group (Fig. 7).

## Discussion

We demonstrated that functional BKCa channels were expressed in WJ-MSCs and that LPS enhances its expression and function of BKCa channels in WJ-MSCs. BKCa channel blockers inhibited WJ-MSC proliferation and induced the apoptosis of these cells. In addition, BKCa channel blockers reduced the level of IL-6 secreted by LPS-stimulated WJ-MSCs. Exosomes secreted by BKCa-knockdown WJ-MSCs exhibited better anti-inflammatory activity, which might be related to the levels of ATP5A1 and filamin B in exosomes.

Previous studies have described the expression of functional BKCa channels in hBMSCs [28], human adipose-derived mesenchymal stem cells (hASCs) [40], and human induced pluripotent stem cell-derived mesenchymal stromal cells (hiPSCs) [41]. However, the expression of BKCa channels in WJ-MSCs has not been reported. BKCa channels play different roles in different types of stem cells. According to Ren et al., BKCa channels in macrophages are activated by LPS, NS1619, and hydrogen sulphide [12]. In the present study, we detected the expression of functional BKCa channels in WJ-MSCs and found that LPS enhanced the expression and opening of BKCa channels in WJ-MSCs, consistent with previous findings in macrophages [12]. BKCa channel inhibition decreases the proliferation of hBMSCs, but promotes the proliferation of hiPSC-MSCs [28, 41]. In the present study, functional BKCa channel blockers inhibited the proliferation of WJ-MSCs, consistent with the findings from hBMSCs.

According to previous studies, BKCa channels play an important role in some inflammatory processes [42, 43]. However, the roles of BKCa channels in different inflammatory responses are not consistent. Researchers have not clearly determined whether BKCa channels alter the immunomodulatory properties of WJ-MSCs. Heparin sulphate (HS) proteoglycans or LPS activate macrophages and induce inflammation by activating Toll-like receptor 4 (TLR4) and BKCa channels, leading to increased IL-6 and TNF-α secretion. The inhibition of BKCa channels reverses p38 phosphorylation and proinflammatory cytokine secretion [12], whereas the activation of BKCa channels inhibits airway inflammation and ameliorates asthma symptoms [14]. The knockout of BKCa channels in smooth muscle increases the mortality rate of mice due to organ injury and septic shock [15]. Our study supports the hypothesis that the inhibition of BKCa channels exerts a beneficial effect on the immune activity of WJ-MSCs. First, BKCa channel blockers inhibited IL-6secreted by LPS-stimulated WJ-MSCs. Second, the study described in the literature and our previous experiment have shown that WJ-MSCs do not express TLR4 [40], indicating that LPS activates BKCa channels in WJ-MSCs through a TLR4-independent mechanism. Therefore, we hypothesized that BKCa channel inhibition might enhance the immunoactivity of WJ-MSCs.

Exosomes derived from MSCs effectively repair oxidative damage in the liver [44], reduce the symptoms of pulmonary oedema caused by endotoxin in mice [45], regulate the proportion of macrophage subtypes during chronic inflammation [46], and promote the immune balance and other inflammatory diseases. IL-6 has pleiotropic effects in inflammation and the immune response. In inflammatory responses, IL-6 promotes the synthesis of acute phase proteins, such as C-reactive protein, serum amyloid A, and fibrinogen in hepatocytes, and inhibits albumin synthesis; therefore, the IL-6 level is often measured in the clinic to assess the severity of inflammation [47]. In addition, IL-6 promotes the production of antibodies by activated B cells, regulates the differentiation of CD4 T cells into Th17 cell subtypes [48], and induces CD8+ T cells to differentiate into cytotoxic T cells [49], thereby mediating innate and adaptive immunity. Moderate level of IL-6 helps the body to repair itself, whereas excess IL-6 production mediates the development of various diseases. IL-10 is considered an anti-inflammatory cytokine [50]. In the present study, exosomes derived from WJ-MSCs after BKCa channel knockdown (si-exo) significantly reduced the secretion of IL-6 by LPS-stimulated THP-1 cells and promoted IL-10 secretion compared with the control conditions. These data indicate that the knockdown of BKCa channels enhances the immunoregulatory activity of WJ-MSC-derived exosomes during acute inflammation.

We conducted a proteomics analysis of MSC exosomes with or without the knockdown of BKCa channel expression to investigate the mechanism by which BKCa channels modulate the immunoregulatory activity of MSCs. The proteomics analysis revealed significantly increased expression of ATP5A1 in the si-exo group compared with the other groups. Previous analyses of proteins interacting with BKCa channels in cochlear cells revealed that BKCa channels interact with ATP synthase, but the specific mechanism remains unclear. ATP5A1 enhances the activity of mitochondrial ATP synthase and reduces the endotoxin-induced inflammatory response of cardiomyocytes [51]. ATP5A1 is also an important target through which the toxoplasma GRA8 antigen stimulates organisms to exhibit bactericidal activity [52]. Moreover, ferritin light chain (FTL), filamin B, and translin levels were reduced in the si-exo group compared with the nc-exo group. Filamin B is a crucial regulator of the transendothelial migration of leukocytes, which promotes the early inflammatory response of endothelial cells [53]. As shown in the study by Bandaru S et al., macrophages lacking filamin A secrete lower levels of the proinflammatory cytokine IL-6 during the process of atherosclerosis [54]. Filamins B and A share a very high level of homology; therefore, we speculate that filamin B may exert similar proinflammatory effects. However, the specific mechanism by which BKCa channels alter protein expression in WJ-MSC exosomes remains unclear. Various studies and proteomic analyses have identified proteins with post-translational modifications (PTM) in exosomes, which mainly include ubiquitinated and phosphorylated proteins, as well as glycosylated proteins and proteins with other modifications. PTMs direct the loading of exosome-enriched proteins by controlling the selective mechanisms of protein cargo sorting, which are useful for determining the prognosis, diagnosis and treatment of diseases [55, 56]. According to Daniele P. et al., palmitoylation of the Alix protein helps stabilize the lipid membrane structure of exosome-like small extracellular vesicles [57]. We were inspired to further explore the effect of BKCa channel activity on the post-transcriptional translation of proteins in exosomes secreted by WJ-MSC.

By performing GO and KEGG pathway analyses, we found that proteins related to lysosomal metabolism, iron metabolism, glycogen and energy metabolism, and oxidative phosphorylation, among other functions, were significantly altered in the si-exo group (Supplementary Figure 1). Lysosomes are involved in the regulation of the inflammatory response and the development of various immune diseases; they exhibit dual functions in regulating the inflammatory response [58]. Decreased intracellular K^+^ concentrations stimulate calcium influx, which mediates lysosome exocytosis and releases the inflammatory factor IL-1β to promote the occurrence of inflammatory reactions. In addition, Feng et al. observed the expression of BKCa channels on the lysosomal membrane and are important and required to maintain the normal function of this membrane [59]. Mitochondrial BKCa channels (mitoBKCa) are located in the mitochondrial inner membrane (IMM), which harbours the electron transport system and the ATP synthase complex [60]. Li et al. reported that the mitoBKCa channel regulates the respiratory rate, mitochondrial depolarization, and reactive oxygen species (ROS) production [61]. Researchers have speculated that mitoBKCa channel activity is an important regulator of mitochondrial function. Therefore, we hypothesize that the knockdown of BKCa channels might improve the anti-inflammatory activity of WJ-MSC-secreted exosomes by regulating lysosomal metabolism and mitochondrial oxidative stress, but further experiments are needed to explore the specific mechanism. We will further explore how BKCa channel knockdown affects the expression of ATP5A1 and filamin B in WJ-MSC-derived exosomes and verify the effect of this modification in other immune diseases. Our findings may provide a more efficient method for cell-free treatment of inflammatory diseases related to the imbalance of multiple cytokines, such as sepsis.

## Conclusions

This study revealed that BKCa channel knockdown can enhance the negative immune regulation of the acute inflammatory response by WJ-MSC-secreted exosomes, mainly by suppressing the secretion of IL-6 by macrophages. As a new type of cell-free therapy, it provides a new method for regulating immune response. Moreover, modified exosomes have the advantages of safety, efficacy, speed, and easy application.

## Supplementary information

**Additional file 1.**

## Data Availability

All the data generated or analysed during this study are included in this article and its additional information files.

## Abbreviations

ATCC: American Type Culture Collection
α-MEM: Minimal essential medium alpha
ATP5A1: Mitochondrial ATP synthase alpha subunit
BKCa channels: Large-conductance Ca^2+^-activated K^+^ channels
BCA: Bicinchoninic acid
CD73: Cluster of differentiation 73
CD90: Cluster of differentiation 90
CD45: Cluster of differentiation 45
DTT: Dithiothreitol
ELISA: Enzyme-linked immunosorbent assays
FITC: Fluorescein isothiocyanate
FBS: Foetal bovine serum
GAPDH: Glyceraldehyde phosphate dehydrogenase
GO: Gene Ontology
hASCs: Human adipose-derived mesenchymal stem cells
hBMSCs: Human bone marrow-derived MSCs
hiPSCs: Human induced pluripotent stem cell-derived mesenchymal stromal cells
HS: Heparin sulphate
IL-1β: Interleukin-1 beta
IL-6: Interleukin-6
IL-10: Interleukin-10
IFN-γ: Interferon γ
IBMX: 3-Isobutyl-1-methylxanthine
IMM: Mitochondrial inner membrane
LPS: Lipopolysaccharide
LC/MS-MS: Liquid chromatography tandem mass spectrometry
MSCs: Mesenchymal stem cells
PMA: Phorbol 12-myristate 13-acetate
PBS: Phosphate-buffered saline
PI: Propidium iodide
PVDF: Polyvinylidene fluoride
real-time PCR: Real-time polymerase chain reaction
SDS-PAGE: Sodium dodecyl sulphate polyacrylamide gel electrophoresis
SP3: Single-pot, solid-phase-enhanced sample-preparation
TBS: Tris-HCl-buffered saline
TLR4: Toll-like receptor 4
THP-1 cell line: Human monocytic cell line
TNF-α: Tumour necrosis factor alpha
WJ-MSCs: Wharton’s jelly-derived mesenchymal stem cells
