# Supplementary figures and images for: BKCa channels regulate the immunomodulatory properties of WJ-MSCs by affecting the exosome protein profiles during the inflammatory response
Source: Stem Cell Res Ther. 2020 Oct 15;11:440. doi: 10.1186/s13287-020-01952-9 (PMC7560248; doi:10.1186/s13287-020-01952-9)

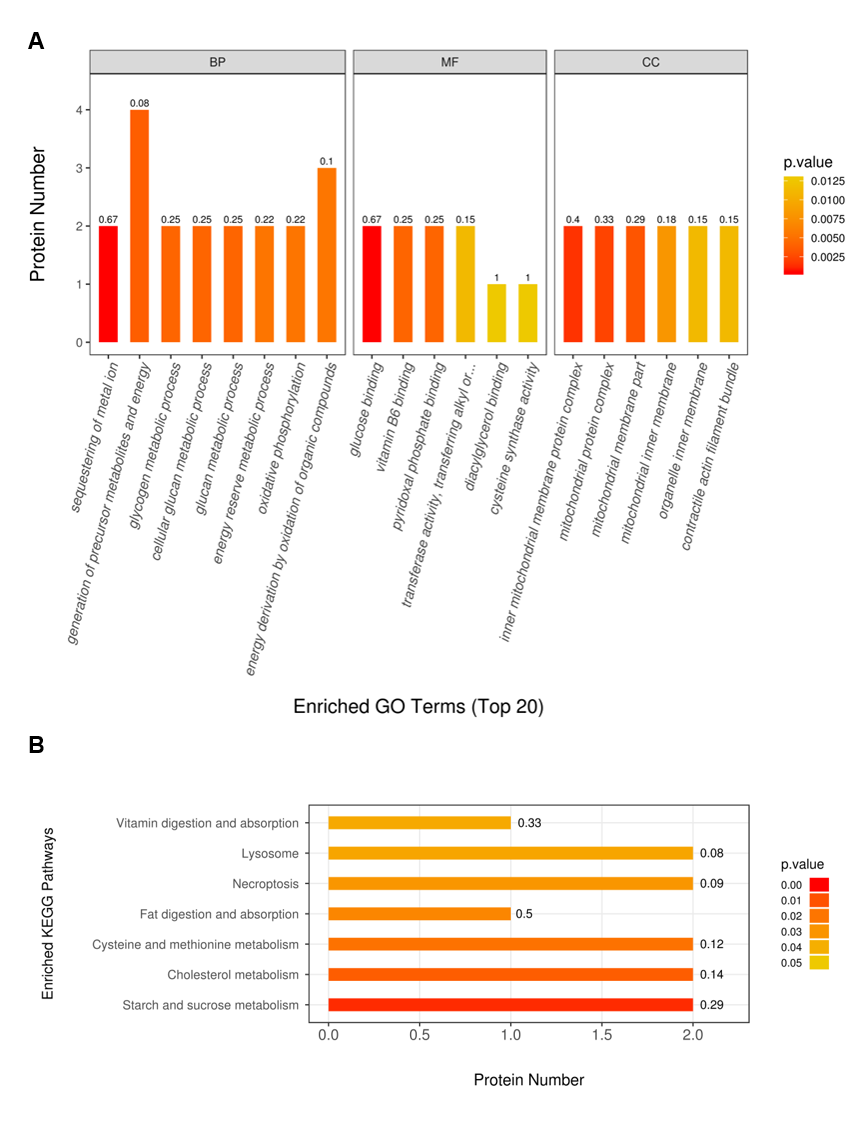

Supplement: Supplementary file 1 — Additional file 1. [file 13287_2020_1952_MOESM1_ESM.png]
